# *QuickStats:* Percentage* of Adults Aged ≥18 Years Who Were Prescribed Medication in the Past 12 Months,^†^ by Sex and Age Group — National Health Interview Survey,^§^ 2017

**DOI:** 10.15585/mmwr.mm6804a6

**Published:** 2019-02-01

**Figure:**
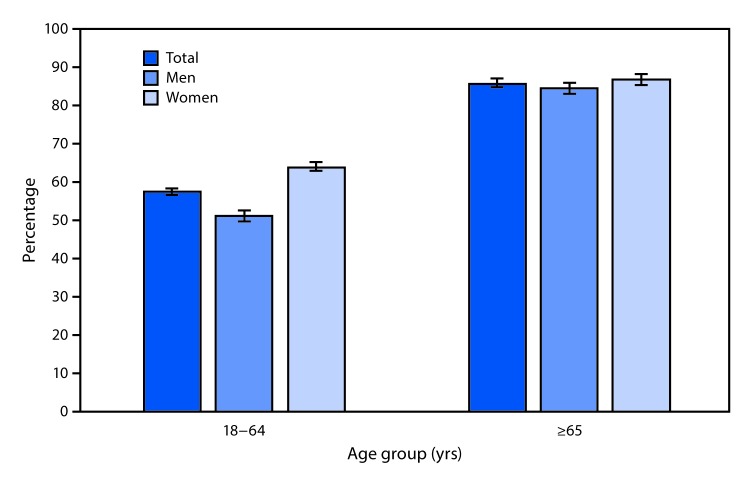
In 2017, 57.9% of adults aged 18–64 years and 86.1% of adults aged ≥65 years were prescribed medication in the past 12 months. Overall and for both men and women separately, receipt of a prescription increased with age. Among both age groups, a greater percentage of women were prescribed medication than men, with 64.3% of women and 51.3% of men aged 18–64 years and 87.1% of women and 85.0% of men aged ≥65 years having been prescribed medication.

**Source:** National Health Interview Survey, 2017. https://www.cdc.gov/nchs/nhis/index.htm.

